# Anti-inflammatory effects of apo-9′-fucoxanthinone from the brown alga, *Sargassum muticum*

**DOI:** 10.1186/2008-2231-21-62

**Published:** 2013-07-26

**Authors:** Eun-Jin Yang, Young Min Ham, Wook Jae Lee, Nam Ho Lee, Chang-Gu Hyun

**Affiliations:** 1 Jeju Biodiversity Research Institute (JBRI), Jeju Technopark, Jeju 699-943, Korea; 2 Department of Chemistry, Cosmetic Science Center, Jeju National University, Jeju 690-756, Korea; 3 LINC Agency, Jeju National University, Ara-1-dong, Jeju 690-756, Korea

**Keywords:** Apo-9′-fucoxanthinone, Brown alga, *Sargassum muticum*, Inflammation

## Abstract

**Background:**

The marine environment is a unique source of bioactive natural products, of which *Sargassum muticum* (Yendo) Fensholt is an important brown algae distributed in Jeju Island, Korea. *S. muticum* is a traditional Korean food stuff and has pharmacological functions including anti-inflammatory effects. However, the active ingredients from *S. muticum* have not been characterized.

**Methods:**

Bioguided fractionation of the ethanolic extract of *S. muticum*, collected from Jeju island, led to the isolation of a norisoprenoid. Its structure was determined by analysis of the spectroscopic data. In vitro anti-inflammatory activity and mechanisms of action of this compound were examined using lipopolysaccharide (LPS)-stimulated RAW 264.7 cells through ELISA assays and Western blot analysis.

**Results:**

Apo-9′-fucoxanthinone, belonging to the norisoprenoid family were identified. Apo-9′-fucoxanthinone effectively suppressed LPS-induced nitric oxide (NO) and prostaglandin E_2_ (PGE_2_) production. This compound also exerted their anti-inflammatory actions by down-regulating of NF-κB activation via suppression of IκB-α in macrophages.

**Conclusions:**

This is the first report describing effective anti-inflammatory activity for apo-9’-fucoxanthinone′-fucoxanthnone isolated from *S. muticum*. Apo-9′-fucoxanthinone may be a good candidate for delaying the progression of human inflammatory diseases and warrants further studies.

## Background

Inflammation is the response of an organism to invasion by foreign pathogens such as parasites, bacteria and viruses. The inflammatory response is an important protective reaction to injury, irritation and infection and is characterised by redness, heat, swelling, loss of function and pain [1]. In the inflammatory state, activated immune cells, such as macrophages secrete large amounts of proinflammatory cytokines, nitric oxide (NO), and prostaglandin E_2_ (PGE_2_). However, high levels of NO and PGE_2_ in a chronic inflammation state can result in various pathological conditions [1-4]. For this reason, regulation of the production of NO and PGE_2_ in macrophages are current research topics for the development of new anti-inflammatory agents. There have been many attempts to derive new anti-inflammatory agents from natural compounds [5-7]. Traditional remedies derived from terrestrial plants and maritime plants such as seaweeds have been considered safe, less toxic, and readily available, even through their modes of action are yet infinite for the most part. Thus, uncovering the molecular mechanism underlying the biological function of natural products might be a good strategy for identifying new therapeutic agents [8,9].

*Sargassum muticum* (Yendo) Fensholt, a brown alga, is the most important economic seaweed, and widely distributed on the seashore of southern and eastern Korea. It is commonly consumed as a popular marine vegetable for more than 1000 years in Korea, particularly in Jeju Island. It has various biological activities, including antioxidant, anti-inflammatory, and antibacterial activities [10,11]. Previously, our research group documented the anti-inflammatory properties of various seaweads [11-14]. During our on-going screening program designed to identify the anti-inflammatory potential of natural compounds, we have isolated apo-9′-fucoxanthinone from *S. muticum*, using activity-directed fractionation, and characterized apo-9′-fucoxanthinone’s structural identity using spectroscopy (^1^H and ^13^NMR) in this study. Also, as a prelude to revealing the anti-inflammatory effects and its mechanisms of apo-9′-fucoxanthinone, the present study focused on whether apo-9′-fucoxanthinone inhibited the production of NO and PGE_2_ and expression of inducible nitric oxide synthase (iNOS) and cyclooxygenase (COX)-2 in LPS-stimulated macrophages.

## Methods

### Extraction and isolation of apo-9′-fucoxanthinone

**Figure 1:**
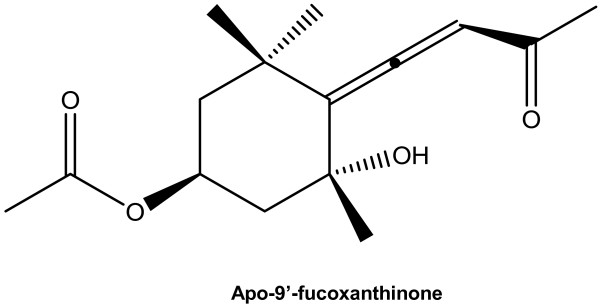
L The structure of Apo-9′-fucoxanthinone.

*S. muticum* was collected from the coasts of Jeju Island in March 2009, and verified by Dr. Wook Jae Lee at Jeju Technopark (JTP). A voucher specimen (CSC-002) was deposited at Department of Chemistry, Jeju National University, Jeju, Korea. *S. muticum* were washed 3 times with water to remove any salt, epiphytes, and sand attached to the surface. They were dried at 60°C for 24 h in an oven, and pulverized in a grinder prior to extraction. The dried powder (800 g) was extracted with 70% aqueous ethanol with stirring for 2 days at room temperature. The filtrate was concentrated under reduced pressure. The extract (105 g) was suspended in water (1.0 L), and successively partitioned into *n*-hexane, methylene chloride, ethyl acetate, and *n*-butanol fractions. The fraction of methylene chloride (7 g), being dissolved in solvent, mixed with celite, and evaporated using a rotary vacuum evaporator. After lyophilization, it was chromatographed and eluted by using the solvents 500 mL of into *n*-hexane, methylene chloride/ethyl acetate (10:1, 5:1, 2:1), methylene chloride, ethyl acetate, and methanol in order. The hexane fraction was chromatographed over a silica gel column using n-hexane:EtOAc (3:1) in order to obtain 10 sub-fractions (F-1 to F-10). All fractions containing the same constituent(s) identified on the TLC plates were combined and the solvents were evaporated using a rotary vacuum evaporator. Structures of fraction 10 of them (F10, 2.3 g) were determined using proton-nuclear magnetic resonance (^1^H NMR) and ^13^C NMR. The compound’s structural identity was determined by one-and two-dimensional nuclear magnetic resonance (NMR) spectroscopic analysis (Additional file 1) and comparison to published values. Structures of these compounds are given in Figure 1.

### Chemicals and reagents

Dulbecco’s modified Eagle’s medium (DMEM) and foetal bovine serum (FBS) were obtained from Invitrogen-Gibco (Grand Island, NY, USA). Enzyme-linked immunosorbent assay (ELISA) kits for prostaglandin E_2_ (PGE_2_) was purchased from R&D Systems, Inc. (St. Louis, MO, USA). Anti-IκB-α, anti-phosphorylated IκB-α (anti-p-IκB-α) were purchased from Cell Signaling Technology (Beverly, MA, USA). Pyrollidine dithiocarbamate (PDTC, a specific inhibitor of NF-κB) was purchased from Calbiochem (San Diego, CA, USA). All other reagents were purchased from Sigma-Aldrich Chemical Co. (St Louis, MO, USA).

### RAW 264.7 cell culture

RAW 264.7 cells were obtained from the Korean Cell Line Bank (KCLB; Seoul, Korea) and maintained at sub-confluence in a 95% air, 5% CO_2_ humidified atmosphere at 37°C as described previously [11-14]. Cells at passages 10–20 were used for the experiments and subcultured every 2–3 days. The medium for routine sub-cultivation was DMEM supplemented with FBS (10%), penicillin (100 units/mL), and streptomycin (100 μg/mL). Cells were counted with a haemocytometer, and the number of viable cells was assessed by trypan blue dye exclusion method.

### MTT assay for cell viability

Cell viability was measured as described previously [11-14] with slight modification using MTT assay. RAW 264.7 cells were cultured in 96-well plates for 18 h, followed by treatment with LPS (1 μg/mL) in the presence of various concentrations of the sample. After a 24-h incubation, MTT was added to the medium for 4 h. Finally, the supernatant was removed and the formazan crystals were dissolved in DMSO. Absorbance was measured at 540 nm. The percentage of cells showing cytotoxicity relative to the control group was determined.

### Nitric oxide determination

RAW 264.7 cells were plated at 1.5 × 10^5^ cells/well in 24-well plates and then incubated with or without LPS (1 μg/mL) in the absence or presence of various concentrations (12.5, 25, 50, and 100 μg/mL) of apo-9-fucoxanthinone for 24 h. Nitrite levels in culture media were determined as described previously [11-14] with slight modification using the Griess reaction and presumed to reflect NO levels. Briefly, the culture supernatant (100 μL) was mixed with the same volume of Griess reagent (1% sulphanilamide and 0.1% N-[1-naphthyl]-ethylenediamine dihydrochloride in 5% phosphoric acid %) for 10 min. Absorbance was the measured at 540 nm using spectrophotometer. Fresh culture media were used as blanks in all experiments. NO levels in samples were read off a standard sodium nitrite curve.

### Detection of PGE_2_ in supernatant

Sandwich ELISA was used to determine the inhibitory effects of various concentrations (12.5, 25, 50, and 100 μg/mL) of apo-9-fucoxanthinone on the production of cytokines PGE_2_ in LPS-treated RAW 264.7 cells. RAW 264.7 cells were stimulated for 24 h before the supernatant was harvested and assayed according to the manufacturer’s protocol for the relevant ELISA kit. Results from 3 independent experiments were used for statistical analysis.

### Western blot analysis

Western blotting was performed with a SDS-PAGE Electrophoresis System as described previously [11-14]. Briefly, the RAW 264.7 cells (5.0 × 10^5^ cells/mL) were pre-incubated for 18 h and then treated with LPS (1 μg/mL) plus aliquots sample for 24 h. After incubation, the cells were washed twice with cold PBS. Whole-cell lysates (25 μg) were separated by 10% sodium dodecyl sulphate-polyacrylamide gel electrophoresis (SDS-PAGE) and electro-transferred to a polyvinylidene fluoride (PVDF) membrane (BIO-RAD, HC, USA). The membrane was incubated for 24 h with 5% skim milk and then incubated with iNOS (1:2500), COX-2 (1:2500), IκB-α (1:1000), phosphorylated IκB-α, antibodies (1:1000) at room temperature for 2 h. The membrane was washed 4 times with TTBS and incubated for 30 min with a peroxidase-conjugated secondary antibody (1:5000) at room temperature. Finally, The immunoactive proteins were detected using an enhanced chemiluminescence (ECL) Western blotting detection kit (Amersharm Pharmacia Biotech., NY, USA).

### Statistical analysis

Results are presented as the means ± standard deviation of at least three replicates. The Student *t*-test was used for statistical analyses of the difference noted. *P* values of 0.05 or less were considered statistically significant.

## Results and discussion

Brown algae have proven to be rich sources of structurally novel and biologically active natural compounds in recent study. These compounds have served as important chemical prototypes for the discovery of new drugs for use in the treatment of various human diseases [15]. Brown algae are also very popular sea vegetables, and many people consider this vegetable as a food of health benefit in East Asia such as Korea, China, and Japan. Jeju Island, the largest island in Korea, is located in the southwest of the Korean Strait, and is well known for its distinctive environment. In particular, the sea levels around this island are known to fluctuate rapidly as a result of global warming. Therefore, in response to this unusual environment, the brown algae that are present on Jeju Island may possess substantial endogenous protective mechanisms [12]. Some studies on brown algae-derived anti-inflammatory compounds have investigated potential inhibitory effects by using the LPS-stimulated murine macrophages [16-18]. Previously, we found that the a *S. muticum* extract displayed an appreciable anti-inflammatory effect in mouse macrophage RAW264.7 cells [11]. In the present study, we isolated the active substance, in an attempt to understand the possible anti-inflammatory mechanism of *S. muticum*. To identify its active components, the ethanol extract was suspended in H_2_O and extracted successively with *n*-hexane, methylene chloride, ethyl acetate, and *n*-butanol. The methylene chloride fraction was subjected repeatedly to column chromatography over celite and silica gel in various solvent systems, to yield the active ingradent. It was identified as apo-9′fucoxanthinone (Figure 1) by comparison of physical and spectroscopic data with published values.

**Figure 2:**
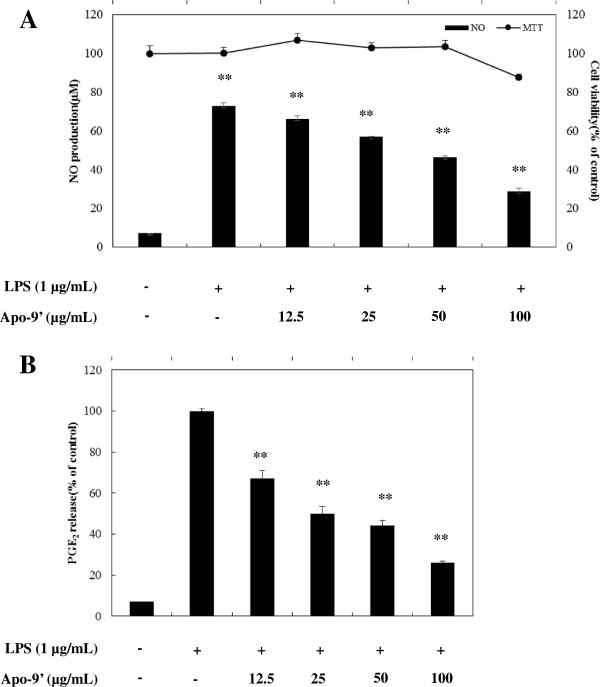
**Effect of Apo-9′-fucoxanthinone on nitric oxide and PGE**_**2 **_**production in LPS-stimulated RAW264.7 cells.** The cells were stimulated with 1 μg/mL of LPS only or with LPS plus various concentrations (12.5, 25, 50, and 100 μg/mL) of Apo-9′ for 24 hr. Nitric oxide production was determined by the Griess reagent method. After a 24-h incubation, PGE_2_ in the culture supernatants was measured by an enzyme-linked immunosorbent assay (ELISA) kit. Cell viability was determined from the 24 hr culture of cells stimulated with LPS (1 μg/mL) in the presence of Apo-9′. The data represent the mean ± SD of triplicate experiments.*P < 0.05, **P < 0.01 versus LPS alone.

**Figure 3:**
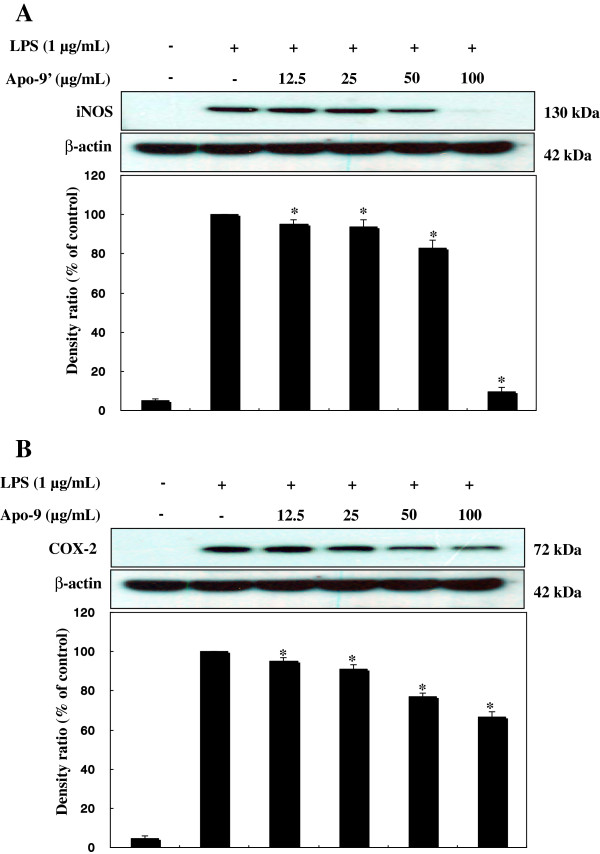
**Effect of Apo-9′-fucoxanthinone on the activation of iNOS and COX-2 in LPS-stimulated RAW 264.7 cells.** RAW 264.7 cells (5.0 × 10^5^ cells/mL) were stimulated with LPS (1 μg/mL) in the Apo-9′ (12.5, 25, 50, and 100 μg/mL) for 24 hr. Whole-cell lysate (25 μg) were prepared and the protein level was subjected to 10% SDS-PAGE, and expression of iNOS, COX-2, and β-actin were determined by Western blotting.

In murine macrophage RAW264.7 cells, LPS alone induces the transcription and protein synthesis of iNOS and COX-2, which produce large amounts of NO and PGE_2_, respectively. Excess production of NO by iNOS has been implicated in a wide spectrum of diseases including septic shock, rheumatoid arthritis, cerebral ischemia, multiple sclerosis, and diabetes [19]. For this reason, NO production induced by LPS through iNOS can reflect the degree of inflammation, and a change in NO level through inhibition of iNOS enzyme activity or iNOS induction provides a means of assessing the effect of agents on the inflammatory process. Therefore, the modulation of macrophage-mediated inflammatory responses is emerging as a promising new therapeutic approach against inflammatory diseases [12-14,20,21]. In an effort to characterize the anti-inflammatory activities of apo-9′fucoxanthinone, we firstly assessed the effects of apo-9′fucoxanthinone on LPS induced NO production in RAW 264.7 cells. Since the half-life of NO is very short, we used nitrite production as an indicator of NO released by LPS-activated macrophages. As shown in Figure 2A, compared to in normal macrophages, NO production increased >15 fold in LPS-activated macrophages. apo-9′-fucoxanthinone reduced LPS-induced NO production in a dose-dependent manner: At apo-9′-fucoxanthinone concentrations of 12.5 μg/mL, 25 μg/mL, 50 μg/mL, and 100 μg/mL, the production of NO by LPS-treated macrophages decreased, as compared with LPS-treated macrophages not treated with apo-9′-fucoxanthinone (Figure 2A). DMSO, the vehicle control, had no effect on NO production (data not shown), reconfirming its immunological inertness. In parallel, the potential cytotoxicity of apo-9′-fucoxanthinone was evaluated by an MTT assay after incubating cells for 24 h in the absence and presence of LPS. However, cell viability was negligibly affected at the concentrations used (12.5 μg/mL, 25 μg/mL, 50 μg/mL, and 100 μg/mL) to inhibit NO (Figure 2A). Thus, the inhibitory effects of apo-9′-fucoxanthinone were not attributable to cytotoxicity. To further elucidate the mechanisms by which apo-9′-fucoxanthinone inhibited NO production in LPS-activated macrophages, we analyzed apo-9′-fucoxanthinone’s effect on LPS-induced iNOS gene expression in macrophages. Under normal conditions, RAW 264.7 cells expressed non-detectable levels of iNOS mRNA, but iNOS mRNA levels increased markedly after 24 h of LPS stimulation (Figure 3A). With the addition of apo-9′-fucoxanthinone (12.5 μg/mL - 100 μg/mL), dose-dependent inhibition of iNOS expression was observed, indicating that apo-9′-fucoxanthinone modulates iNOS expression.

PGE_2_ is an inflammatory mediator that is produced from the conversion of arachidonic acid by cyclooxygenase. In a variety of inflammatory cells, including macrophages, COX-2 is induced by cytokines and other activators, such as LPS, resulting in the release of a large amount of PGE_2_ at inflammatory sites. Numerous studies have reported that prostaglandin (PGE)_2_ participate in inflammatory and nociceptive events [22-24]. Therefore its ubiquitous role in the pathogenesis of inflammatory gene expression, PGE_2_ is a current target for treating various diseases. For this reason, we next examined the effects of apo-9′-fucoxanthinone on PGE_2_ production in LPS-stimulated RAW 264.7 macrophages. Cells were pre-incubated with apo-9′-fucoxanthinone for 1 h, following which they were stimulated with 1 μg/mL LPS for 24 h. As shown in Figure 2B, Compared to unstimulated macrophages, the PGE_2_ level increased dramatically by 15-fold in LPS-stimulated macrophages. With the addition of apo-9′-fucoxanthinone (12.5 μg/mL, 25 μg/mL, 50 μg/mL, and 100 μg/mL) a dose-dependent reduction in PGE_2_ was observed (Figure 2B). In order to determine the mechanism by which apo-9′-fucoxanthinone reduces LPS-induced PGE_2_ production, we studied the ability of apo-9′-fucoxanthinone to influence the LPS-induced expression of COX-2. The addition of LPS resulted in a clearly defined increase in COX-2 expression that was markedly attenuated in a dose-dependent fashion when treated with apo-9′-fucoxanthinone (Figure 3B), corroborating that apo-9′-fucoxanthinone induces a decrease in COX-2, which translates into a dramatic decrease in PGE_2_.

**Figure 4:**
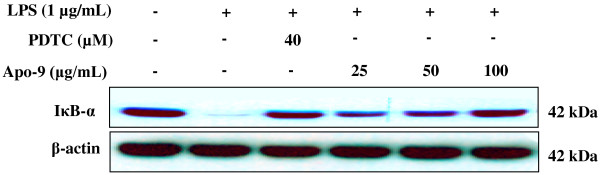
**Effects of Apo-9′-fucoxanthinone on the degradation of IκB-α in LPS stimulated RAW 264.7 cells.** RAW 264.7 cells (1.0 × 10^6^ cells/mL) were stimulated with LPS (1 μg/mL) in the presence of apo-9′-fucoxanthinone (12.5, 25, 50, and 100 μg/mL) or PDTC (40 μM) for 15 min. Whole cell lysates (30 ug) were prepared and the protein level was subjected to 12% SDS-PAGE, and expression of IkB-α and β-actin were determined by Western blotting. The β-actin antibody as a loading control.

NF-κB activation, in response to pro-inflammatory stimuli, involves the rapid phosphorylation of IκBs by the IKK signalosome complex. Free NF-κB produced by this process translocates to the nucleus, where it binds to κB-binding sites in the promoter regions of target genes. It then induces the transcription of pro-inflammatory mediators such as iNOS and COX-2. Actually, several studies have shown that anti-inflammatory agents inhibit the activation of NF-κB by preventing IκB degradation [25-27]. Thus, we attempted in this study to determine whether or not apo-9′-fucoxanthinone inhibits the phosphorylation and degradation of IκB. Accordingly, RAW 264.7 cells were pretreated for 30 min with 9′fucoxanthinone, and IκB-α protein levels were determined after 15 min of further LPS exposure (1 μg/mL). As shown in Figure 4, apo-9′-fucoxanthinone was shown to significantly suppress the LPS-induced IκB-α degradation. As expected, the reference compounds 2-amino-4-methyl pyridine (iNOS inhibitor) also potently inhibited the LPS-induced IκB-α degradation at 40 μM. These results show that apo-9′-fucoxanthinone inhibits LPS induced NF-κB activation by preventing the IκB-α degradation.

## Conclusions

The results of this study reveal, for the first time, that the apo-9′-fucoxanthinone isolated from *S. muticum* exhibit anti-inflammatory properties through suppressing NO and PGE_2_ production in LPS-stimulated RAW 264.7 cells by attenuation of NF-κB-mediated iNOS and COX-2 expression. It is proposed that that apo-9′-fucoxanthinone is a potential anti-inflammatory agent and may be used in the future to treat inflammation-associated human health. To our knowledge, this is the first report concerning the evaluation of the anti-inflammatory properties of apo-9′-fucoxanthinone.

## Competing interests

The authors declare that they have no competing interests.

## Authors’ contributions

EJY carried out the anti-inflammatory evaluation. YMH carried out the isolation and purification apo-9’-fucoxanthinone. WJL carried out the preparation and identification of alga material. NHL carried out the interpretation of the NMR data and identification of the compounds. CGH conceived of the study, and participated in its design and coordination and helped to draft the manuscript. All authors read and approved the final manuscript.

## Supplementary Material

Additional file 1 Apo-9'-fucoxanthinone. Click here for file
