# Supplementary material for: Anti-inflammatory effects of apo-9′-fucoxanthinone from the brown alga, Sargassum muticum
Source: Daru. 2013 Jul 26;21(1):62. doi: 10.1186/2008-2231-21-62 (PMC3733608; doi:10.1186/2008-2231-21-62)

[Apo-9'-fucoxanthinone]

a colorless oily phase; positive ESIMS m/z $(C_{15}H_{22}O_4Na)^+$289.16; ^{1}H-NMR (500 MHz, $CDCl_3$) δ: 1.13 (3H, s, H-12), 1.43 (6H, s, H-11 and 13), 1.44 (1H, m, H-2a), 1.52 (dd, J = 12.5 and 11.5 Hz, H-4b), 1.99 (1H, m, H-2b), 2.02 (3H, s, H-15), 2.16 (3H, s, H-10), 2.32 (ddd, J = 2.0, 4.0 and 13.0 Hz, H-4a), 5.38 (1H, tt, J = 11.5 and 4.5 Hz, H-3), 5.84 (1H, s, H-8); ^{13}C-NMR (125MHz, $CDCl_3$) δ: 21.30 (C-15), 26.37 (C-10), 28.90 (C-11), 30.78 (C-13), 31.59 (C-12), 35.99 (C-1), 44.97 (C-2), 44.02 (C-4), 67.41 (C-3), 71.98 (C-5), 100.86 (C-8), 118.42(C-6), 170.35 (C-14), 198.02 (C-9), 209.49 (C-7).

Apo-9'-fucoxanthinone

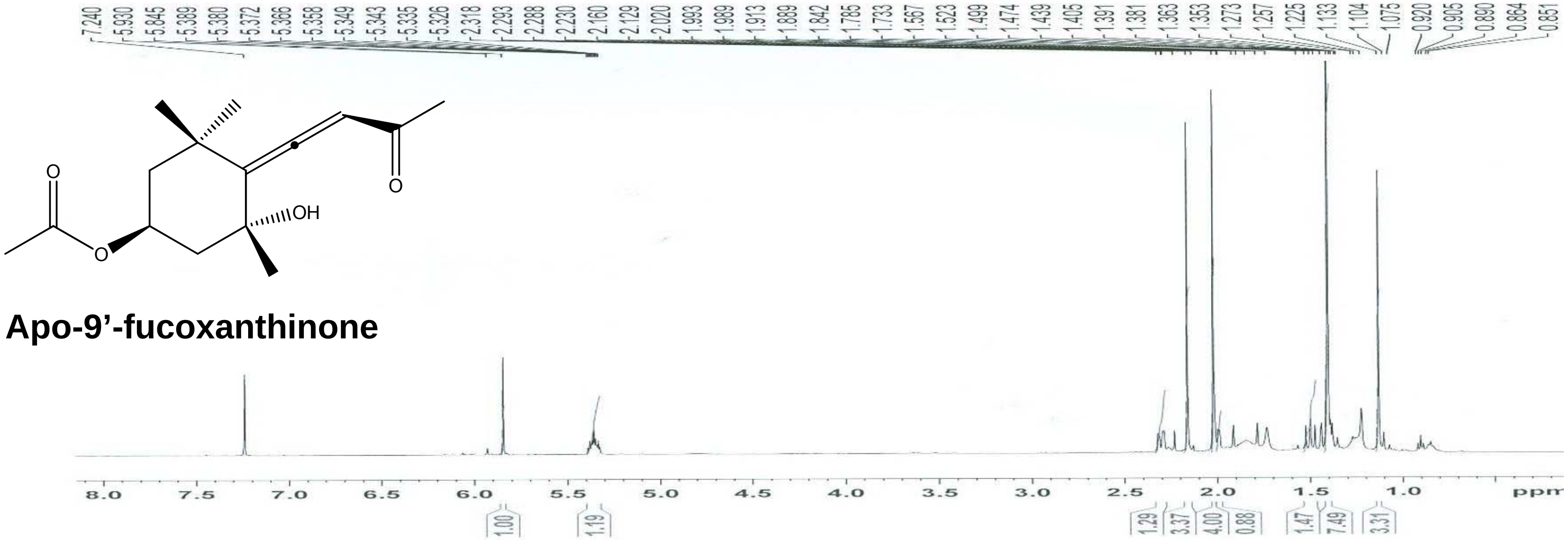

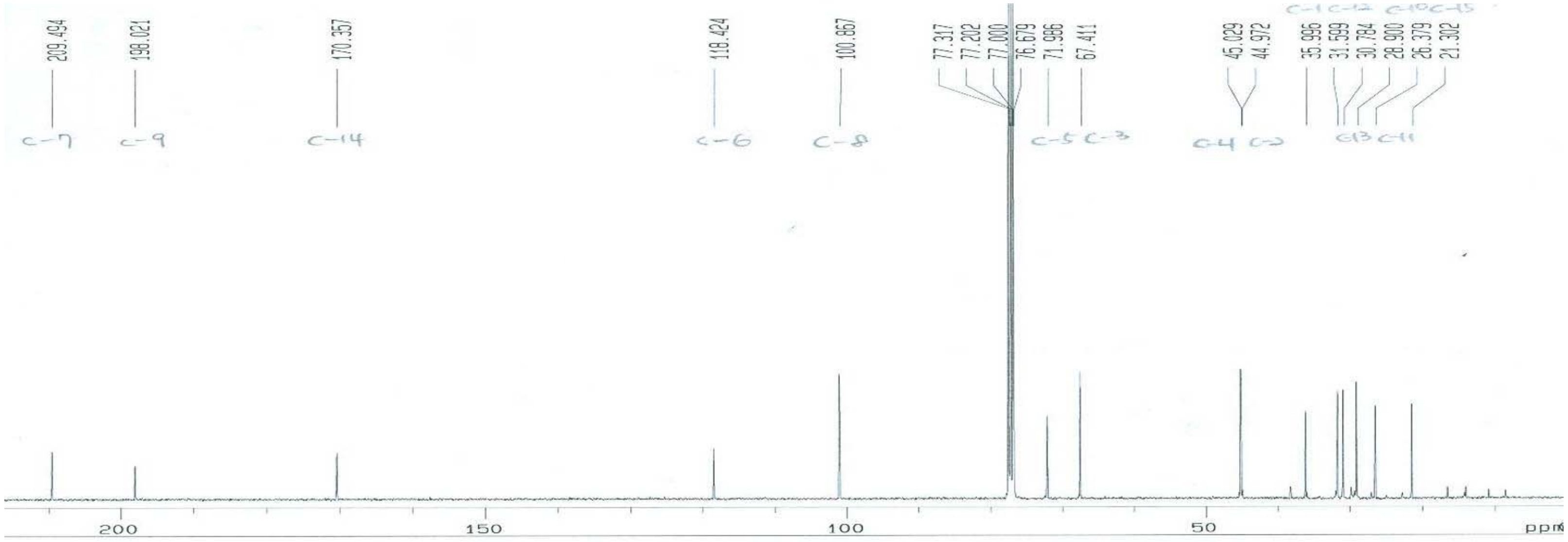

Apo-9'-fucoxanthinone LC-MS spectrum

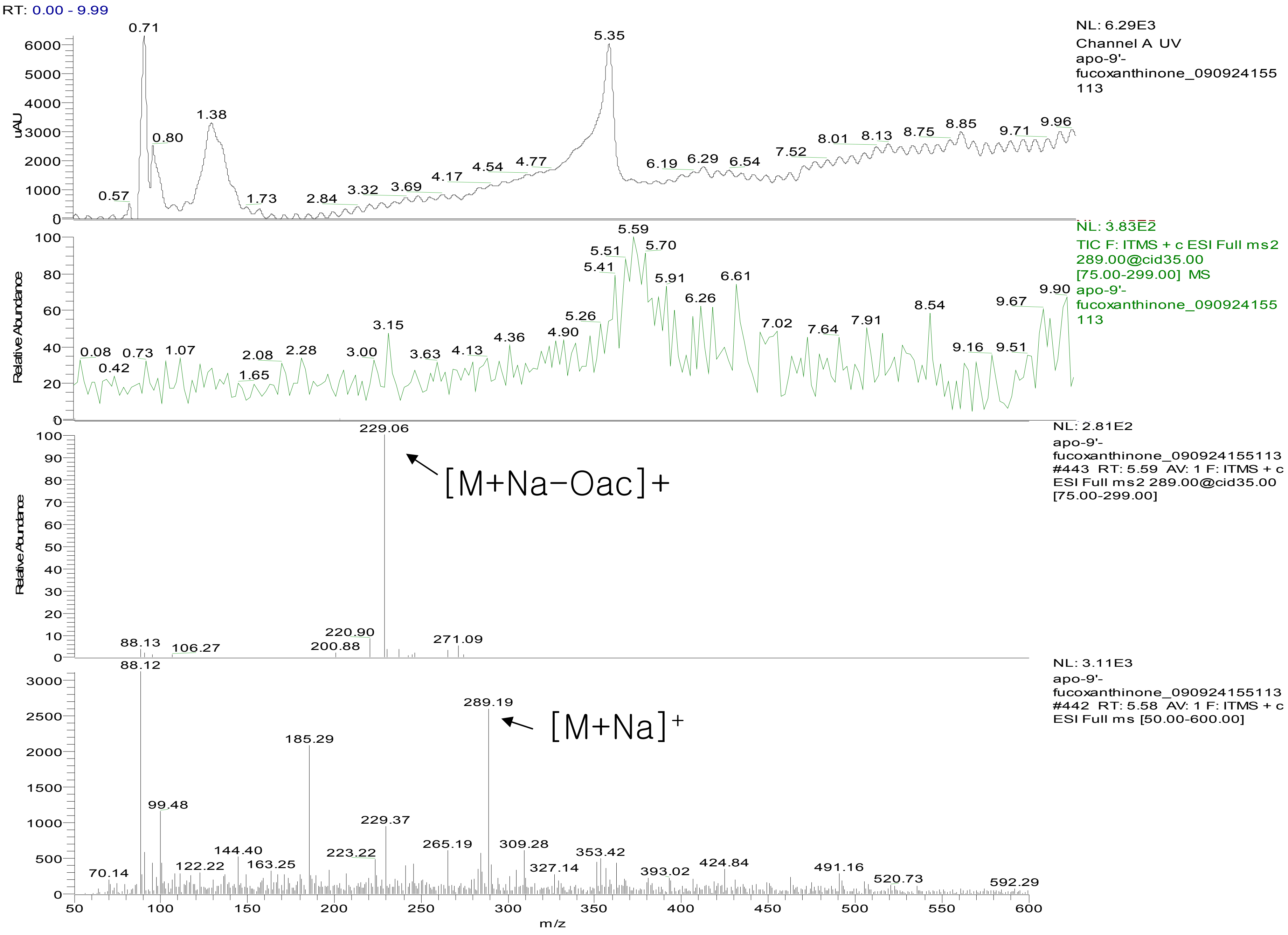

Supplement: Additional file 1 — Apo-9'-fucoxanthinone. [file 2008-2231-21-62-S1.pdf]
